# THE ROLE OF DEMENTIA AND SERIOUS ILLNESS IN RECEIPT OF PAID CARE

**DOI:** 10.1093/geroni/igad104.1197

**Published:** 2023-12-21

**Authors:** Jennifer Reckrey, Hannah Kleijwegt, Amy Kelley, Katherine Ornstein

**Affiliations:** Icahn School of Medicine at Mount Sinai, New York City, New York, United States; Icahn School of Medicine at Mount Sinai, New York City, New York, United States; National Institute on Aging, National Institutes of Health, Bethesda, Maryland, United States; Johns Hopkins University, Baltimore, Maryland, United States

## Abstract

Little is known about receipt of paid care for individuals living at home with dementia and other non-dementia serious illnesses. This retrospective cohort study sought to characterize receipt of paid care among individuals with impairment in self-care activities and/or other manifestations of serious illness in the context of family caregiving and socioeconomic status. Data from participants ≥65 years enrolled in the Health and Retirement Study between 1998-2018 with new-onset need for help in ≥1 self-care task was linked to fee-for-service Medicare claims (sample n=2,521). We found that those who received paid care experienced higher healthcare utilization than those without paid care. Receipt of paid care was highest among those with multiple manifestations of serious illness: 41.7% of those with self-care impairment, dementia, and non-dementia serious illness received ≥40 hours of paid care per week. When present, paid care accounted for 54% of total caregiving hours. In multivariable models, those with Medicaid were more likely to receive paid care (p<0.001), but those in the highest income quartile received more hours of paid care (p=0.05.) Those with non-dementia serious illness were more likely to receive paid care (p<0.001), but those with dementia received more hours of paid care (p<0.001). The role of paid caregivers grows as people experience multiple manifestations of serious illness and high care hours are common among those with dementia. Future work should explore how paid caregivers can collaborate with families and healthcare teams to improve the health and well-being of the seriously ill living at home.

